# Standardized Protocol for Comprehensive, Non-Invasive Phenotyping of Atrial Myopathy in Sprague-Dawley Rat Models of Metabolic Syndrome Using Clinical-Grade Echocardiography and Electrophysiology Systems

**DOI:** 10.3390/mps9040103

**Published:** 2026-07-02

**Authors:** Ardian Rizal, Mohammad Saifur Rohman, Fatchiyah Fatchiyah, Hidayat Sujuti, Anna Fuji Rahimah, Wella Karolina, Victor Alvianoes Guterez Hose, Mokhammad Afifudin

**Affiliations:** 1 Doctoral Program in Medical Science, Faculty of Medicine, Universitas Brawijaya, Malang 65145, East Java, Indonesia; fatchiya@ub.ac.id (F.F.); hidayatsujuti.fk@ub.ac.id (H.S.); 2 Department of Cardiology and Vascular Medicine, Faculty of Medicine, Universitas Brawijaya, Dr. Saiful Anwar General Hospital, Malang 65111, East Java, Indonesia; drannafuji@ub.ac.id (A.F.R.); wella.karolina@ub.ac.id (W.K.); dr_afif_cardio@student.ub.ac.id (M.A.); 3 Cardiovascular Research Centre, Faculty of Medicine, Universitas Brawijaya, Malang 65145, East Java, Indonesia; victorhose345@student.ub.ac.id; 4 Research Centre of Smart Molecule of Natural Resource, Universitas Brawijaya, Malang 65145, East Java, Indonesia; 5 Departement of Ophthalmology, Faculty of Medicine, Universitas Brawijaya, Dr. Saiful Anwar General Hospital, Malang 65111, East Java, Indonesia

**Keywords:** atrial myopathy, atrial fibrillation, metabolic syndrome, echocardiography, transesophageal pacing, animal model protocol

## Abstract

**Background:** Small animal models are essential for atrial fibrillation (AF) research. Researchers in AF use an electrocardiogram (ECG), echocardiography and invasive electrophysiology study (EPS) to assess atrial structural and electrical remodeling. In relatively smaller cardiac structures and rapid heart rates, the examination can be challenging without special tools designed for animal study. Moreover, conventional invasive EPSs often cause significant trauma, alter autonomic tone, and limit longitudinal evaluations. This study aimed to evaluate the feasibility of repurposing hospital-grade medical devices for the non-invasive, multi-modality assessment of atrial myopathy in a rat model of metabolic syndrome (MetS). **Methods:** A total of 12 male Sprague-Dawley rats underwent the multi-modality assessment. Structural remodeling was evaluated using hospital-grade echocardiography (8–12 MHz) to measure left atrial (LA) dimensions and volume. Surface ECG was used to determine P-wave duration. Electrical remodeling and AF inducibility were assessed using transesophageal pacing (TEP)-based EPS, evaluating the atrial effective refractory period (AERP), sinus node recovery time (SNRT), and response to rapid atrial burst pacing. **Results:** The protocols showed high procedural safety (survival rate 91.67%) and successfully characterized atrial myopathy. Surface ECG showed marked intra-atrial conduction delay with prolonged P-wave duration in the MetS group (30.17 ± 4.62 vs. 22.33 ± 1.86 ms, *p* < 0.05). Echocardiography revealed signs of structural remodeling in the MetS group, evidenced by marked prolonged Isovolumic Relaxation Time (IVRT: 35.602 ± 3.043 vs. 19.187 ± 3.631 ms; *p* < 0.001) and increased Left Atrial Area (0.223 ± 0.0556 vs. 0.134 ± 0.033; *p* = 0.007). Furthermore, TEP-based EPS quantified electrical remodeling. The MetS group had shorter AERP (73.33 ± 10.33 ms vs. 120.00 ± 34.06 ms; *p* = 0.010) and Corrected SNRT (100.67 ± 53.98 ms) versus controls (208.33 ± 76.97 ms; *p* = 0.018). The MetS group exhibited a higher absolute AF inducibility rate (50%, three out of six rats) compared to the SH group (33.3%, two out of six rats). **Conclusions:** The integration of surface ECG, echocardiography, and TEP-based EPS provides a safe, highly reproducible, and comprehensive method for evaluating both structural and electrical components of atrial myopathy in small animal models, allowing for robust longitudinal studies.

## 1. Introduction

Atrial fibrillation (AF) remains the most prevalent cardiac arrhythmia globally, contributing significantly to cardiovascular morbidity [1]. Robust basic science research using reliable in vivo small animal models has become indispensable for understanding knowledge gaps [2], specifically, the early progression of structural and electrical remodeling known as atrial myopathy. A comprehensive evaluation of this arrhythmogenic substrate requires the assessment of structural dilation (via echocardiography) and electrical vulnerability, such as the shortening of the atrial effective refractory period (AERP) and AF inducibility (via electrophysiology studies) [3].

However, accurately phenotyping this arrhythmogenic substrate in small rodents presents severe technical and financial challenges. Current contemporary guidelines strongly recommend the use of ultra-high-frequency micro-ultrasound systems (utilizing 30–40 MHz transducers) to accommodate the extremely small cardiac dimensions and rapid resting heart rates (300–400 bpm) of rodents [4]. Similarly, electrophysiological (EP) assessments traditionally depend on custom-built, animal-specific electrical stimulators and micro-recording systems [5]. While these specialized laboratory devices provide high-resolution data, they are prohibitively expensive, severely restricting accessibility for many research centers and creating a bottleneck in cardiovascular translational research.

Furthermore, traditional EP studies in rodent models heavily rely on highly invasive methodologies, such as open-chest epicardial pacing or the insertion of intracardiac catheters via jugular vein cutdowns [6]. These invasive surgical approaches possess critical limitations: the associated surgical trauma and the requirement for deep anesthesia significantly depress and alter the intrinsic cardiac autonomic tone and creating less physiologic environment [2]. Additionally, the destructive nature of these procedures results in high peri-procedural mortality, eliminating the need for long-term, longitudinal evaluations in the same subjects.

To bridge this fundamental translational gap, there is an urgent need to develop and validate standardized protocols that repurpose universally accessible, hospital-grade diagnostic tools for small animal research. Standard clinical echocardiography systems equipped with relatively lower frequency phased-array probes (8–12 MHz), typically used for pediatric or neonatal patients, and standard human EP mapping systems (e.g., Claris Workmate) are ubiquitous in hospital catheterization laboratories. Yet, they are rarely utilized in basic science due to the lack of established technical protocols to overcome the spatial and temporal resolutions required for rodent hearts.

Therefore, this study aims to describe, optimize, and validate a comprehensive, non-invasive step-by-step protocol integrating surface electrocardiography (ECG), hospital-grade echocardiography, and transesophageal pacing (TEP)-based EP studies (EPSs) by utilizing a metabolic syndrome (MetS) rat model. The strategic selection of a metabolic syndrome (MetS) rat model in this methodological study serves as a rigorous proof-of-concept for our non-invasive setup. Unlike other cardiac injury models—such as myocardial infarction—which induce gross, acute, and easily detectable macrostructural destruction, MetS triggers a subtle, slow, and diffuse form of atrial myopathy. Consequently, demonstrating that our optimized protocol using repurposed clinical-grade devices can accurately capture and quantify these conservative, early-stage structural and electrical variations establishes the high sensitivity and precision of the system. Furthermore, because MetS is a chronic pathology requiring an extended timeline (16 weeks), it perfectly underscores the unique advantage of our low-mortality, non-invasive modalities, which effectively eliminate the procedural fatalities of traditional open-chest epicardial surgeries and safely permit long-term, longitudinal phenotyping within the exact same subjects over time [7].

## 2. Experimental Design

### 2.1. Materials

- Animals: 12 male Sprague-Dawley rats (aged 8 weeks at the start of induction).
- Diet: High-Fat High-Sucrose (HFHS) diet for MetS induction according to a previous publication, consisting of mixing a powdered normal pellet diet (250 g/kg), sucrose (300 g/kg), hydrogenated vegetable fat (200 g/kg), egg yolks (200 g/kg), salt (25 g/kg), monosodium glutamate (22 g/kg), and DL-methionine (3 g/kg) [8].
- Chemicals: Streptozotocin (STZ) (Sigma-Aldrich, St. Louis, MO, USA).
- Anesthesia: Ketamine hydrochloride 100 mg/mL (Ket-A-100, Peru) combined with xylazine 20 mg/mL (Interchemie, Venray, The Netherlands).
- Consumables: Supreme™ Electrophysiology Diagnostic Catheter 4Fr (Abbot Medical, originally St Jude Medical).

### 2.2. Equipment

- WorkMate Claris™ System (Abbot Medical, St. Paul, MN, USA).
- Vivid™ iq Ultrasound System (GE Health Care, Chicago, IL, USA).
- Probe/Transducer 12S-RS (GE Health Care, Chicago, IL, USA).

## 3. Procedure

### 3.1. Animal Preparation and MetS Induction (Time for Completion: 16 Weeks)

- Divide the rats into two cohorts: a healthy control group (n = 6) and a MetS group (n = 6).
- Induce the MetS phenotype by administering an HFHS diet formula to the intervention group ad libitum for 16 consecutive weeks, while the control group receives standard normal rodent pellet diet.
- At the end of the second week of dietary manipulation, administer a single low dose of Streptozotocin (STZ) 30 mg/kg body weight, dissolved in 0.01 M citrate buffer (pH 4.5), via a single intraperitoneal (i.p.) injection to the HFHS cohort. The control group receives an equivalent i.p. volume of 0.01 M citrate buffer alone to mimic procedural stress.
- Verify the successful establishment of the clinical MetS phenotype in week 8 and week 16 by measuring physiological and biochemical markers. Fasting blood glucose (FBG) is analyzed via tail-vein pricking after a 12 h fast using a calibrated handheld glucometer (Accu-Chek Performa, Roche, Germany). Lipid profiles, including triglycerides (TG) and high-density lipoprotein cholesterol (HDL-C), are quantified from blood serum using standard enzymatic colorimetric assays on an automated clinical chemistry analyzer (Cobas c311, Roche Diagnostics Corporation, Indianapolis, IN, USA).

### 3.2. Anesthesia and Subject Positioning (Time for Completion: 5–10 Min)

- Induction and Maintenance: Ketamine hydrochloride combined with xylazine.
- Dosage: Ketamine 75 mg/kg and xylazine 10 mg/kg body weight.
- Route of Administration: Single intraperitoneal injection.
- Once the loss of righting reflex is confirmed, transfer the rat to a temperature-controlled heating platform.
- The depth of anesthesia should be strictly monitored and minimized to prevent the artificial depression of atrial function and heart rate.

⚠ CRITICAL STEP Strict monitoring of anesthesia depth is paramount. Deep anesthesia artificially depresses intrinsic autonomic tone and heart rate, and may cause fatality and non-physiological conditions that alter echocardiographic and electrophysiological measurements.

### 3.3. Protocol 1: Echocardiography Assessment for Structural Remodeling (Time for Completion: 15–20 Min)

1. Shave the pre-cordial region and apply warmed acoustic gel.
2. Place the rat in a supine or slight left lateral decubitus position.
3. Image acquisition and optimalization: Adjust the 2D frame rate to >100 frames per second (fps) by narrowing the sector width and minimizing the imaging depth (restricted to 2–3 cm) to encompass only the region of interest.
4. Acquire the Parasternal Long Axis (PSLAX) view. Place an M-mode cursor perpendicular to the LV walls just below the mitral valve to measure the interventricular septum thickness (IVSd) and LV dimensions (LVIDd and LVIDs).

⚠ CRITICAL STEP Set the M-mode sweep speed to its maximum (150–200 mm/s) to accurately capture the rapid end-systolic and end-diastolic phases associated with the high physiological rodent heart rate (>300 bpm).

5. Atrial Structure Measurement: Measure the left atrial diameter (LAD) using M-Mode at the end of ventricular systole (maximal filling phase). From the Apical 4-Chamber (A4C) equivalent view, trace the left atrial volume (LAV) or area (LAA) using the Area-Length method.
6. Diastolic Function (IVRT and transmitral flow velocity): From the Apical 4-Chamber (A4C) equivalent view, activate Pulsed-Wave (PW) Doppler. Place the sample volume precisely at the tips of the mitral valve leaflets. Record and measure the peak early (E) and late (A) transmitral diastolic velocities and IVRT.
7. Tissue Doppler Imaging (TDI) for Mitral Annular Velocity (e’): While maintaining the optimized A4C view, activate the Tissue Doppler Imaging (TDI) modality. Place the PW Doppler sample volume at the septal aspect of the mitral annulus. Record the cardiac cycle and measure the peak early diastolic annular velocity (e’).

### 3.4. Protocol 2: Transesophageal Pacing (TEP) and Electrophysiology Study (Time for Completion: 30–45 Min)

**Figure 1:**
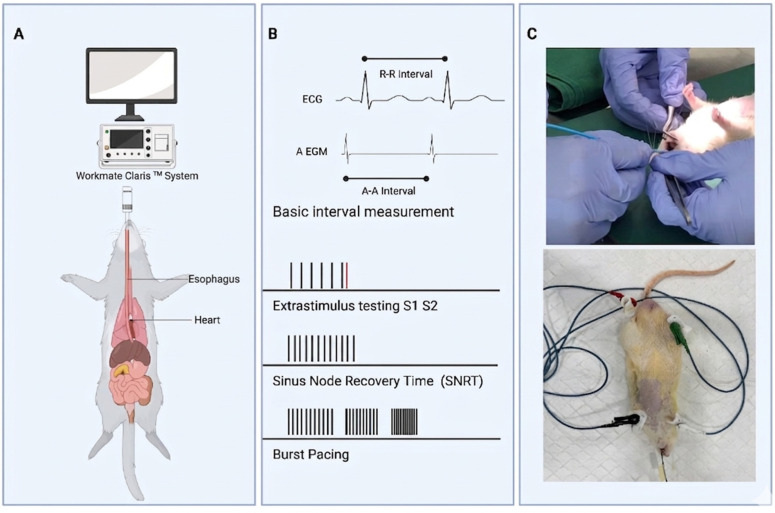
Electrophysiology study. (**A**) Schematic anatomical sagittal plane demonstrating the non-invasive placement of the 4 Fr quadripolar catheter within the esophagus, in close proximity to the left atrium. (**B**) Graphical schematics of the programmed electrical stimulation protocols, including basic intervals, S1–S2 extrastimulus testing for AERP, overdrive pacing for SNRT, and rapid atrial burst pacing for AF induction. (**C**) Sequential procedural photographs for technical replication.

**Figure 2:**
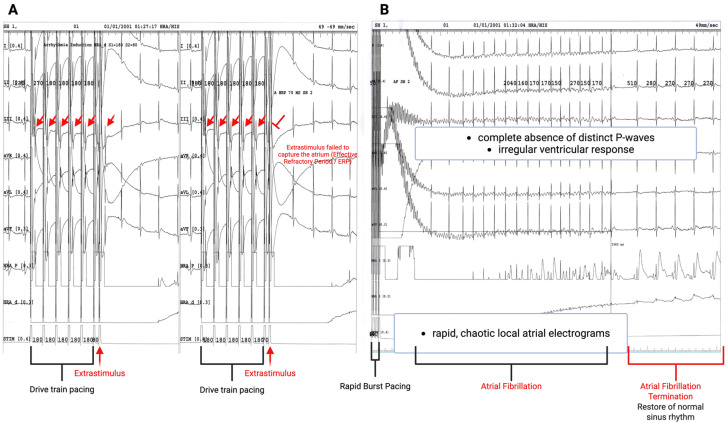
Electrophysiology study results. (**A**) Programmed electrical stimulation using a standard S1–S2 extra stimulus protocol to determine the atrial effective refractory period (AERP). The pacing spike is clearly followed by local atrial capture (red arrow). (**B**) An aggressive burst pacing protocol successfully triggers a rapid, chaotic, and irregular atrial rhythm, demonstrating absolute atrial fibrillation (AF).

This protocol details the non-invasive quantification of the arrhythmogenic substrate using the Abbott Claris Workmate EP System (Figure 1 and Figure 2).

1. Catheter Insertion: Insert a 4 Fr quadripolar diagnostic catheter orally into the esophagus.
2. Optimal Positioning: Advance the catheter (typically 3.5 to 4.5 cm from the incisors) while monitoring the bipolar esophageal electrogram in real time on the Claris Workmate monitor. Define optimal placement as the depth yielding the sharpest and highest amplitude A-wave, confirming anatomical proximity to the left atrium.
3. Signal Optimization and Troubleshooting Artifacts.

⚠ CRITICAL STEP Pacing artifacts frequently obscure immediate post-pacing ECG signals in rodents. (1) Minimum pacing output: Optimize the signal-to-noise ratio by strictly using the minimum pacing output (mA) required for capture. (2) Amplifier blanking adjustment: Enable stimulus blanking features on the Claris Console (20ms). (3) Bandpass filtering: Apply specific high-pass and low-pass band filters (30–500 Hz) on the Workmate console to minimize baseline wander and sharpen the local atrial deflection.

4. Diastolic Pacing Threshold: Initiate pacing at a basic cycle length (200 ms or 20 ms faster than the basic rhythm). Start current output at a minimal level (0.5 mA) and incrementally increase until consistent 1:1 atrial capture is achieved. Set operational output at 1.5–2 times this threshold to prevent vagal stimulation.
5. Atrial effective refractory period (AERP): Deliver a programmed electrical stimulation (S1–S2) protocol. Following an eight-stimulus drive train (S1), deliver a premature extra stimulus (S2) starting 150–140 ms. Decrease S2 in 10 ms decrements until the S2 stimulus fails to capture an atrial response. Record the longest failing S1–S2 interval as the AERP.
6. Sinus Node Function: Evaluate the corrected sinus node recovery time (CSNRT) by measuring the interval from the last pacing spike of an overdrive burst to the first spontaneous sinus return cycle, subtracting the basic cycle length.
7. AF Inducibility Protocol: Deliver modified rapid atrial burst pacing protocol. Burst trains lasting 5 to 15 s, using various cycle lengths (100, 80, 50, 40, 30, 20, and 10 ms). Define positive AF induction as the complete loss of distinct P-waves accompanied, presence of rapid chaotic local atrial electrograms (EGM) and irregular ventricular response lasting for more than 2 s [9,10].

⚠ CRITICAL STEP Differentiate true atrial capture from AV nodal Wenckebach. Rely on the local esophageal A-signal to confirm atrial capture, while using the surface QRS to evaluate ventricular conduction. A dropped QRS with a preserved esophageal A-signal confirms physiological AV block, not loss of pacing capture.

### 3.5. Statistical Analysis and Sample Size Justification

The sample size for this study was initially determined using the minimum sample size formula for animal experimental designs, adopting the framework established by Federer Formula. Based on this standard calculation, a sample size of n = 6 animals per group was selected to balance statistical requirements with the ethical principles of the 3Rs (Replacement, Reduction, and Refinement). It is central to note that this investigation was primarily designed and optimized as a methodological feasibility and protocol validation study, rather than a fully powered biological efficacy trial.

Continuous variables are presented as mean and standard deviation (SD). Statistical significance for normally distributed continuous data was determined using the Independent Student’s *t*-test, while the Mann–Whitney U test was applied for non-parametric continuous variables. To retrospectively validate the adequacy of our cohort size for this protocol, a post hoc power analysis was performed upon study completion using the observed means and standard deviations of the primary continuous endpoints. A *p*-value < 0.05 was considered statistically significant. All statistical analyses were performed using GraphPad Prism version 9.0.

## 4. Expected Results and Protocol Validation

### 4.1. Procedural Safety and Sample Size Validation

Across two groups, the procedures including anesthesia, surface ECG, echocardiography, and TEP-based EPS were well-tolerated. Immediately after the anesthesia procedure, baseline heart rate was comparable between groups. Only 1 (MetS group) out of 12 rats developed severe bradycardia and asystole following the aggressive rapid atrial burst pacing protocol, resulting in a 91.67% survival rate. There were no incidences of esophageal perforation or fatal bleeding. All remaining subjects could complete all the examinations and fully recover, and were followed up until day 5 after the procedure without any mortality found.

To confirm that the sample size of n=6 per group provided sufficient statistical rigor to validate this protocol, a post hoc power analysis was done. Based on the distinct variations and large biological effect sizes observed in our continuous datasets (Cohen’s d≥1.8), the retrospective analysis demonstrated an observed statistical power (1−β) of 88.4% for left atrial (LA) area and 92.1% for P-wave duration at a two-tailed alpha (α) level of 0.05. This mathematically confirms that the sample size had enough power to detect macrostructural and electrophysiological differences, minimizing the risk of Type II statistical errors.

### 4.2. Diagnosis Confirmation of Metabolic Syndrome According to Current Guidelines

To confirm the success of disease modeling prior to advanced cardiac phenotyping, a targeted evaluation of biochemical and anthropometric parameters was executed in week 16. According to the original National Cholesterol Education Program Adult Treatment Panel III (NCEP ATP III) guidelines, a clinical diagnosis of MetS requires the presence of at least three out of five specific components [11]. As detailed in Table 1, the intervention cohort successfully fulfilled the diagnostic threshold by showing higher fasting plasma glucose, marked hypertriglyceridemia, and a lower level of cardioprotective high-density lipoprotein cholesterol (HDL-C).

Blood pressure measurement was intentionally excluded from this diagnostic framework to preserve autonomic stability prior to electrophysiological phenotyping. Non-invasive blood pressure measurement in awake rodents via tail-cuff plethysmography requires physical restraint and thermal warming, which induces acute procedural stress and sympathetic catecholamine surges that would artificially distort highly sensitive electrophysiological parameters, such as the atrial effective refractory period (AERP).

### 4.3. Surface ECG Markers

Baseline heart rates after the anesthesia procedure were comparable between groups. The MetS group showed a slower base rate at 238.50 ± 37.86 ms compared to the control group at 206.20 ± 7.76 ms but it was statistically not significant (*p* = 0.096). Compared to the healthy control group, the MetS group showed significant P-wave duration prolongation compared to the healthy controls (30.17 ± 4.62 ms vs. 22.33 ± 1.86 ms; *p* < 0.05).

### 4.4. Echocardiographic Performance and Structural Remodeling

**Figure 3:**
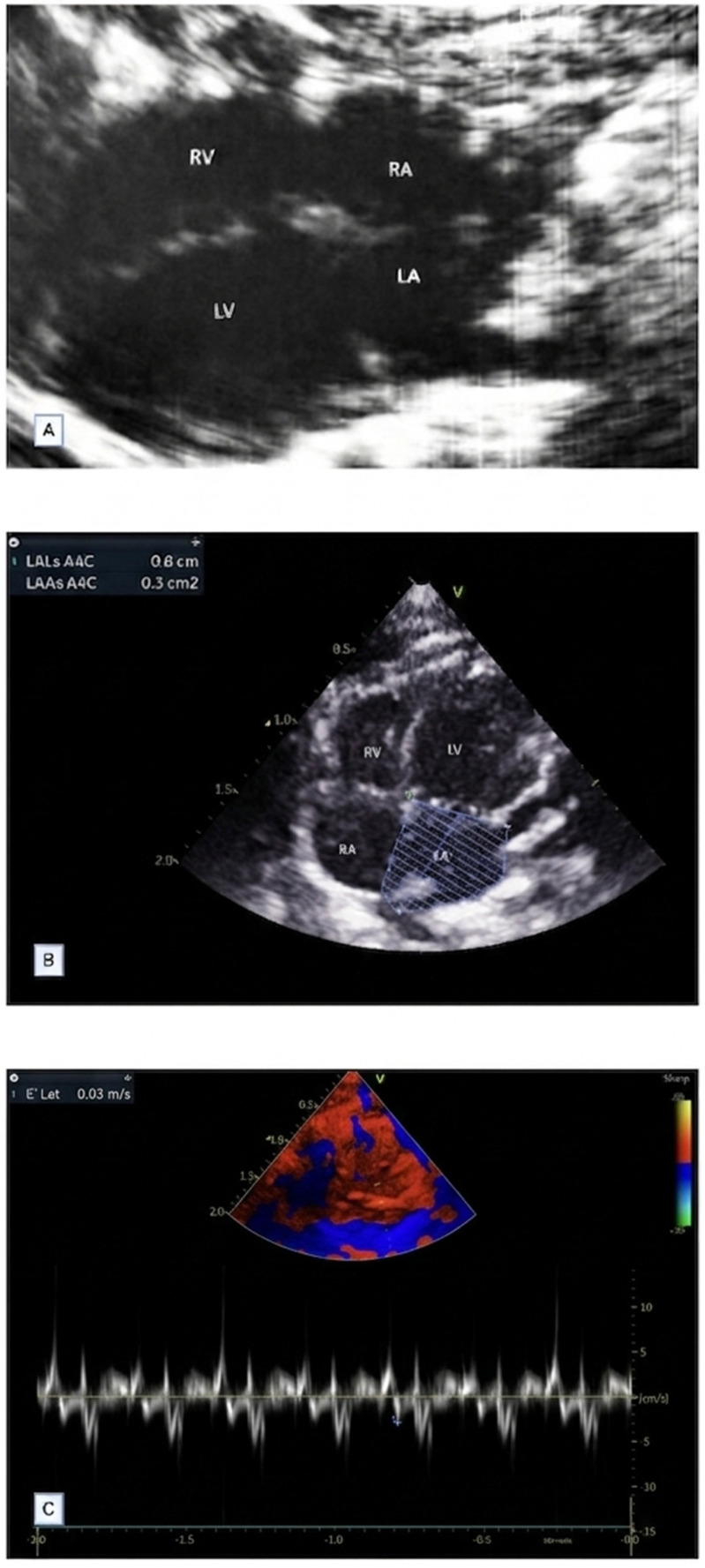
Echocardiography assessment. (**A**) Basic 4 chamber view. (**B**) LA area measurement. (**C**) E lateral.

To ensure the reliability of utilizing a clinical ultrasound system (GE Vivid q) for rodent hearts, we first evaluated the inter-observer agreement. Echocardiographic measurements (Figure 3) obtained from two independent diagnostic assessments demonstrated excellent concordance, yielding a high Intraclass Correlation Coefficient (ICC = 0.89), validating the reproducibility of our repurposed clinical setup.

The baseline echocardiographic parameters of the healthy control group were compared to the standardized reference values for adult rodents established by the ESC Working Group on Myocardial Function [4]. The baseline LV dimension, LV function and diastolic function in our anesthetized control group were physiologically comparable to the established standard references for healthy rodents, confirming the accuracy of our image acquisition protocol (Table 2).

In the context of disease characterization, our echocardiography results successfully captured structural remodeling indicative of atrial myopathy in the metabolic syndrome model in terms of dimension and function (Table 3). The MetS group had significant left atrial dilation compared to the control group. The left atrial length was significantly enlarged in MetS rats (0.560 ± 0.071 cm) compared to healthy controls (0.404 ± 0.062 cm; *p* = 0.005). Concurrently, the left atrial area more than doubled in the MetS group (0.223 ± 0.0556 vs. 0.134 ± 0.033; *p* = 0.007). Atrial myopathy signs were also seen in the LA function parameters, depicted indirectly by diastolic function and LA filling pressure. The MetS group showed significantly higher IVRT values (19.187 ± 3.631 vs. 35.602 ± 3.043, *p* < 0.001) and higher E/e’ (22.892 ± 7.027 vs. 21.207 ± 4.408) although statistically not significant (*p* = 0.638).

### 4.5. Electrophysiological Vulnerability and AF Inducibility

The MetS group demonstrated electrical vulnerability, a hallmark of electrical remodeling prone to atrial myopathy and atrial fibrillation. The atrial effective refractory period (AERP) was notably shortened in the MetS group compared to the healthy control group (73.33 ± 10.33 ms vs. 120.00 ± 34.06 ms; *p* = 0.010). Additionally, the sinus node recovery time (SNRT) showed a trend of alteration as early sign of myopathy. The corrected sinus node recovery time (CSNRT) was significantly shortened in the MetS group (100.67 ± 53.98 ms) versus controls (208.33 ± 76.97 ms; *p* = 0.018). This functional impairment was highly reproducible upon retest measurements within the same procedure (CSNRT 2: 120.67 ± 25.51 ms in MetS vs. 215.33 ± 82.65 ms in control group; *p* = 0.023) as shown in Table 3.

Although a trend towards higher AF inducibility was observed in the MetS group (50% vs. 33.3%), this difference did not reach statistical significance (*p* > 0.99), likely due to the limited sample size.

## 5. Discussion

### 5.1. Principal Findings and Methodological Translation

This study successfully demonstrates the feasibility, safety, and performance of repurposed hospital-grade medical devices for the comprehensive, non-invasive assessment of atrial myopathy in a small rodent model. This approach overcomes the dependency on expensive, animal-exclusive micro-devices and highly invasive open-chest surgeries, thereby offering a highly translatable and accessible for longitudinal cardiovascular research.

### 5.2. Validation of the Clinical-Grade Setup Against Gold Standards

As a major strength of our research, we validated our findings against standardized values of echo parameters according to European ESC guidelines for rodent echocardiography [4] (Table 2). The baseline LV dimensions and functional measurements obtained from our healthy control group were practically identical to the references for ketamine–xylazine-anesthetized rats. Even though minor dimensional variances were observed, specifically a smaller baseline LVIDd and higher LV mass, these are physiologically expected variations strongly attributable to specific local sub-strain characteristics and our strict maintenance of a lighter anesthetic plane. The narrower LVIDd is driven by our lighter anesthetic plane which avoids artificial chamber dilation, while the elevated LV mass reflects an age- and body-weight-dependent adaptation of our older Sprague-Dawley cohort. Furthermore, minor differences in spatial resolution and boundary thickness might be attributed to the lower frequency range of our repurposed clinical transducer (8–12 MHz) compared to the standard ultra-high micro-ultrasound systems (30–40 MHz) typically referenced.

### 5.3. Disease Characterization for MetS Condition

Metabolic syndrome has been established as an independent risk factor of atrial myopathy and atrial fibrillation [12]. Chronic systemic inflammation is a major pathway in MetS-associated complications, including AF. It directly increases oxidative stress and profibrotic signaling within the myocardium. One of the earliest mechanical manifestations of this cascade is left ventricular stiffness, or diastolic dysfunction. As ventricular compliance decreases, diastolic filling pressures inevitably rise, imposing a direct mechanical burden on the left atrium. Because of the nature of the thin-walled atrium, it responds with compensatory dilation. This chronic stretch and structural dilation mark the macroscopic onset of atrial myopathy, a condition that closely associated with the development of AF [3].

This structural remodeling subsequently triggers electrical remodeling [3]. Chronic mechanical stretch and the infiltration of fibrotic scar tissue into the atrial myocardium disrupt intercellular architecture and gap junction integrity, culminating in electrical dyssynchrony and conduction slowing. Furthermore, this tissue-level remodeling alters ion channel function, which clinically manifests as the shortening of the atrial effective refractory period (AERP) [13]. The combination of slowed conduction velocity and a shortened refractory period creates an ideal arrhythmogenic substrate [14]. This specific environment drastically reduces the required wavelength for re-entry, allowing multiple chaotic wavelets to sustain themselves within the dilated atrial chambers, thereby initiating and perpetuating episodes of atrial fibrillation.

The disease characterization phase of our study remarkably replicated this entire pathophysiological process from systemic MetS induction to AF vulnerability. The initial phase of ventricular stiffness was sensitively captured by prolonged IVRT in the MetS group, which was followed by structural dilation of the left atrium. In our study, it was depicted by prolonged p wave duration (seen on ECG) and increased LA area (seen on echocardiography). Hallmarks of electrical remodeling caused by the direct effects of ion channels and the disruption in intracellular calcium dynamics were confirmed by the lower value of AERP and marked sinus node dysfunction (altered CSNRT) during intracardiac pacing [15].

**Figure 4:**
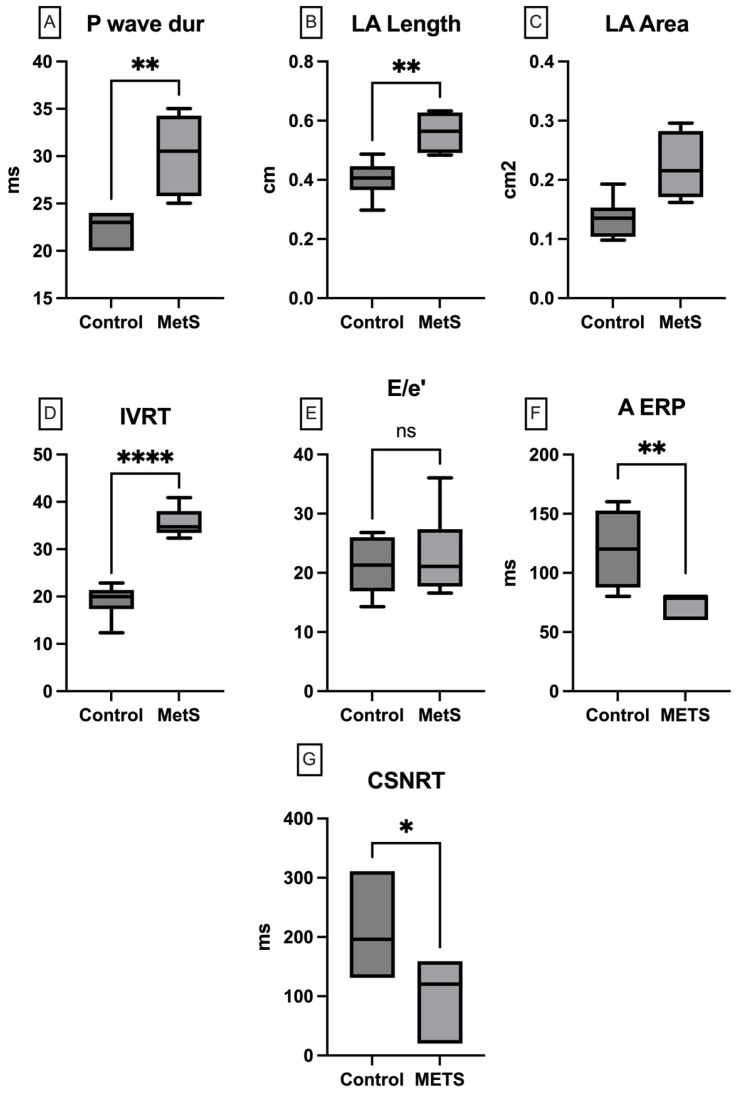
Structural and electrophysiological remodeling in the metabolic syndrome (MetS) rat model. (**A**) P wave duration (ms). (**B**) Left atrial (LA) length (cm). (**C**) LA area (cm^2^). (**D**) Isovolumic relaxation time (IVRT, ms). (**E**) E/e′ ratio (an echocardiographic index of LV diastolic filling pressure). (**F**) Atrial effective refractory period (A ERP, ms). (**G**) Corrected sinus node recovery time (CSNRT, ms). *Data are presented as mean ± SD with individual data points (n = 6 per group)*. * *p* < 0.05, ** *p* < 0.01, and **** *p* < 0.001 vs. *control*.

Regarding the “ultimate” arrhythmic outcome, absolute AF inducibility during burst pacing showed a higher trend in the MetS group but did not reach statistical significance. Beyond the limitation of a small sample size (six per group) which underpowers binary/categorical outcome analyses, these findings (Figure 4) strongly aligns with the physiological constraints of small rodent models. According to the ‘critical mass hypothesis’, sustaining multiple re-entrant wavelets requires a sufficiently large atrial tissue volume to maintain AF [16]. Furthermore, the duration of MetS induction in this model was sufficient to initiate functional electrical remodeling and early compensatory dilation, but perhaps not long enough to develop the dense, irreversible fibrotic architecture required for the initiation and perpetuation of AF [17].

Beyond the MetS paradigm described here, this method has potential to be applied in broad cardiovascular models. Due to its high spatial–temporal precision and minimal procedural mortality, this setup can be readily applied to track the serial macrostructural and electrophysiological progression of atrial myopathy in models of hypertension, obesity, and natural aging—where early diastolic stretch transitions into arrhythmogenic triggers. Furthermore, it serves as a reasonable method for evaluating upstream left atrial volume and structural remodeling in advanced disease states such as heart failure with preserved ejection fraction (HFpEF) and post-myocardial infarction ventricular remodeling, enabling robust, long-term longitudinal drug-screening trials without the systemic confounding factors of destructive open-chest procedures.

## 6. Study Limitations

We acknowledge that a limitation of this study is the relatively small sample size (n = 6 animals per group). However, because this work was primarily designed as a technical feasibility and protocol validation study rather than a powered biological investigation, a retrospective post hoc power analysis was executed to validate this cohort. For continuous variables (Echo and EP parameters), it indicated that this method had enough power. Conversely, this sample size remains statistically underpowered for binary categorical endpoints, which explains why the absolute atrial fibrillation (AF) inducibility rates did not reach statistical significance (*p* > 0.99) despite a clear percentage trend.

A technical limitation for repurposing clinical ultrasound systems is the restriction on evaluating advanced measurement, specifically Global Longitudinal Strain (GLS) or Atrial Strain via Speckle Tracking Echocardiography (STE). Accurate STE in rodent models requires exceptionally high temporal resolution (frame rates typically exceeding 200–300 frames per second) to match their rapid physiological heart rates (>300 bpm) without signal aliasing. Consequently, we relied on classical, highly reproducible functional parameters to successfully characterize the functional evaluation of the atrium.

## 7. Conclusions

In conclusion, the integration of standard surface ECG, hospital-grade echocardiography, and transesophageal pacing-based electrophysiology provides a safe, highly reproducible, and comprehensive platform for evaluating atrial myopathy in small rodent models. This repurposed clinical setup accurately unmasks the pathophysiological cascade of MetS—from diastolic stiffness to profound structural dilation and electrical arrhythmogenic vulnerability—thereby democratizing advanced cardiovascular basic science research without the dependency on prohibitively expensive animal-exclusive devices.

## Figures and Tables

**Table 1:** Validation of metabolic syndrome models.

Parameters	Control Group (n = 6) SI Unit (mmol/L)	MetS Group (n = 6) SI Unit (mmol/L)	*p*-Value	MetS Group mg/dL	NCEP ATP III Status
Fasting Blood Glucose	5.58 ± 0.44	13.43 ± 6.60	0.033	241.7 ± 118.8	Positive (>126 mg/dL)
Triglycerides (TG)	1.02 ± 0.57	1.70 ± 0.42	0.043	150.5 ± 37.1	Positive (>150 mg/dL)
HDL-Cholesterol	1.07 ± 0.18	0.71 ± 0.14	0.004	27.4 ± 5.4	Positive (<40 mg/dL)
Waist Circumference (cm)	14.67 ± 0.82	15.17 ± 0.82	0.314	15.17 ± 0.82	Normal Trajectory

**Table 2:** Validation of baseline echocardiographic parameters in healthy rats compared to ESC reference standards [4].

Parameters	ESC Reference [4] (Mean ± sd)	Control Group (Mean ± sd)	Agreement
**LV Structure**			
IVSD (mm)	1.58 ± 0.05	1.56 ± 0.21	excellent
LVPWD (mm)	1.60 ± 0.23	1.77 ± 0.45	excellent
LVIDD (mm)	6.73 ± 0.43	4.79 ± 0.82	smaller cavity
LV mass (mg)	745 ± 30	955 ± 74	higher mass
**LV Systolic Function**			
EF (%)	79.9 ± 6.3	83.2 ± 5.6	excellent (concordant)
FS	47.8 ± 7.7	46.8 ± 6.1	excellent (identical)
**DIASTOLIC FUNCTION**			
E velocity (mm/s)	741 ± 155	669 ± 99	good
A velocity (mm/s)	454 ± 73	330 ± 89	good
E/A ratio	1.73 ± 0.40	2.15 ± 0.73	concordant (normal diastolic)

**Table 3:** ECG, Echocardiography and EPS parameters of atrial myopathy.

Parameters	Control Group (n = 6)	MetS Group (n = 6)	*p*-Value
**ECG**			
P-wave duration (ms)	22.33 ± 1.86	30.17 ± 4.62	0.005
**Echocardiography**			
LA length (cm)	0.404 ± 0.062	0.560 ± 0.071	0.002
LA area (cm^2^)	0.134 ± 0.033	0.223 ± 0.0556	0.007
E/e’ average	21.207 ± 4.408	22.892 ± 7.027	0.638
IVRT (ms)	19.187 ± 3.631	35.602 ± 3.043	<0.001
**EP Study**			
AERP (ms)	120.00 ± 34.06	73.33 ± 10.33	0.010
CSNRT 1 (ms)	208.33 ± 76.97	100.67 ± 53.98	0.019
CSNRT 2 (ms)	215.33 ± 82.65	120.67 ± 25.51	0.023
AF Inducibility, n (%)	2 (33.3%)	3 (50.0%)	>0.99

Values are presented as mean ± standard deviation (SD) for continuous parameters, and as n (%) for the categorical parameter. *p* < 0.05 indicates a statistically significant difference between the control and MetS groups. Statistical significance was determined using the Independent Student’s *t*-test for normally distributed continuous variables (CSNRT) and the Mann–Whitney U test for non-parametric continuous variables (P-wave duration and AERP). AF inducibility was analyzed using Fisher’s Exact Test. AERP = Atrial Effective Refractory Period; SNRT = Sinus Node Recovery Time; CSNRT = Corrected Sinus Node Recovery Time.

## Data Availability

The raw data supporting the conclusions of this article will be made available by the authors on request.

## Abbreviations

The following abbreviations are used in this manuscript:

A4C	Apical 4-Chamber
AERP	Atrial Effective Refractory Period
AF	Atrial Fibrillation
AV	Atrioventricular
CSNRT	Corrected Sinus Node Recovery Time
ECG	Electrocardiogram
EF	Ejection Fraction
EP	Electrophysiology
EPS	Electrophysiology Study
ESC	European Society of Cardiology
FS	Fractional Shortening
ICC	Intraclass Correlation Coefficient
IVRT	Isovolumic Relaxation Time
IVSd	Interventricular Septal Thickness at End-Diastole
LA	Left Atrial/Left Atrium
LAA	Left Atrial Area
LAD	Left Atrial Diameter
LAV	Left Atrial Volume
LV	Left Ventricle/Left Ventricular
LVIDd	Left Ventricular Internal Diameter at End-Diastole
LVIDs	Left Ventricular Internal Diameter at End-Systole
LVPWD	Left Ventricular Posterior Wall Thickness at End-Diastole
MetS	Metabolic Syndrome
PSLAX	Parasternal Long Axis
SD	Standard Deviation
SNRT	Sinus Node Recovery Time
TEP	Transesophageal Pacing
